# Hypocretin: a promising target for the regulation of homeostasis

**DOI:** 10.3389/fnins.2025.1638270

**Published:** 2025-08-25

**Authors:** Yutong Wang, Su Fu, Jian Mao, Kun Cui, Hong Jiang

**Affiliations:** ^1^Beijing Life Science Academy, Beijing, China; ^2^Department of Neurobiology, School of Basic Medical Sciences, Neuroscience Research Institute, Peking University, Beijing, China

**Keywords:** hypocretin (orexin), sleep, wakefulness, metabolism, mood disorders, narcolepsy

## Abstract

Hypocretin, also known as orexin, is a hypothalamic neuropeptide that regulates essential physiological processes including arousal, energy metabolism, feeding behavior, and emotional states. Through widespread projections and two G-protein-coupled receptors—HCRT-1R and HCRT-2R—the hypocretin system exerts diverse modulatory effects across the central nervous system. The role of hypocretin in maintaining wakefulness is well established, particularly in narcolepsy type 1 (NT1), where loss of hypocretin neurons leads to excessive daytime sleepiness and cataplexy. However, the mechanisms by which hypocretin stabilizes transitions between sleep stages remain incompletely understood. Additionally, while hypocretin integrates metabolic signals such as glucose, leptin, and ghrelin to promote feeding and energy expenditure, NT1 patients paradoxically experience weight gain despite reduced caloric intake—highlighting unresolved questions about hypocretin’s role in energy homeostasis. In the affective domain, preclinical studies suggest hypocretin enhances stress resilience and modulates anxiety- and depression-related behaviors. Yet, human data remain inconsistent, in part due to methodological variability and the limited availability of cerebrospinal fluid sampling to accurately assess central hypocretin function. Therapeutically, the hypocretin system is a promising target across several domains. Dual hypocretin receptor antagonists (DORAs), such as suvorexant and daridorexant, are clinically approved for insomnia. Selective HCRT-2R agonists—including TAK-861 and ALKS-2680—are in clinical trials for NT1 and show encouraging results. Additionally, HCRT-2R antagonists like seltorexant are being explored for major depressive disorder. This review will highlight the anatomical distribution, receptor mechanisms, and physiological functions of the hypocretin system. It will also focus to discuss its role in narcolepsy, metabolic regulation, and mood disorders, while addressing key challenges and open questions that must be resolved to fully harness hypocretin’s therapeutic potential.

## Introduction of hypocretin and its receptors

Hypocretin, also known as Orexin, is a neuropeptide that plays a crucial role in various physiological functions, including the regulation of the sleep/wake cycle, modulation of feeding behavior, and maintenance of energy balance. These peptides are derived from a common precursor polypeptide, prepro-hypocretin, which is encoded by a hypothalamus-specific mRNA, named *prepro-hypocretin* mRNA. The hypocretin system consists of two peptides, Hcrt1 and Hcrt2, also referred to as Orexin A and Orexin B, respectively (de Lecea et al., 1998; Sakurai et al., 1998). Hcrt1 is a 33-amino acid peptide of 3,562 Da, featuring a pyroglutamyl residue at the N-terminus and C-terminal amidation, whereas Hcrt2 is a 28-amino acid, also C-terminally amidated, with peptide of 2,937 Da.

The significance of these peptides became evident in 1998 when de Lecea and Sakurai independently identified them and introduced the term Hypocretin-Orexin Neurons (HONs). This terminology has since been widely adopted to describe the unique neuropeptide system responsible for diverse physiological functions.

Anatomically, hypocretin neurons are predominantly localized in the lateral hypothalamus and posterior hypothalamus, with extensive projections throughout the brain, encompassing regions such as the cortex, olfactory bulb, amygdala, septum, thalamus, locus coeruleus (LC), periaqueductal gray (PAG), and raphe nuclei (de Lecea et al., 1998; Sakurai et al., 1998). At the molecular level, hypocretin exerts its functions through two seven-transmembrane G-protein coupled receptors (GPCRs), namely hypocretin receptor-1 (Hcrt-1R) and hypocretin receptor-2 (Hcrt-2R). Notably, Hcrt-1R displays a higher affinity for Hcrt1-approximately three times greater than that of Hcrt-2R-making it the more selective receptor. In contrast, Hcrt-2R serves as a non-selective receptor that responds to both Hcrt1 and Hcrt2 (Sakurai et al., 1998). Moreover, hypocretin initially binds to hypocretin receptors, activating three subtypes of G proteins (Gq/11, Gi/o, Gs), which subsequently modulate phospholipases, ion channels, and protein kinases, ultimately triggering a variety of downstream signaling pathways (Kukkonen, 2016; Li et al., 2017).

Hcrt-1R and Hcrt-2R are extensively expressed in the central nervous system, with their distribution largely consistent with the projection range of hypocretin neurons. Specifically, Hcrt-1R is predominantly localized in the PAG, LC, and Hcrt-2R is mainly found in the cerebral cortex, ventromedial hypothalamic nucleus (VMH), dorsomedial hypothalamic nucleus (DMH), and other areas. Notably, both subtypes of receptors are expressed by the serotonergic neurons in the median raphe nucleus (MR) and dorsal raphe nucleus (DR) (Marcus et al., 2001; Mitsukawa and Kimura, 2022; Mir et al., 2024).

Hypocretin neurons have extensive projections and their receptors are broadly expressed throughout the brain. This anatomical structure allows the hypocretin system to orchestrate an array of complex physiological processes. In this review, we summarize the anatomical distribution of hypocretin projections and receptors, describe how hypocretin regulates the sleep–wake cycle, feeding, and mood, and highlight recent advances in therapeutically targeting this system.

## The projections of hypocretin neurons and the distribution of hypocretin receptors

**Figure 1:**
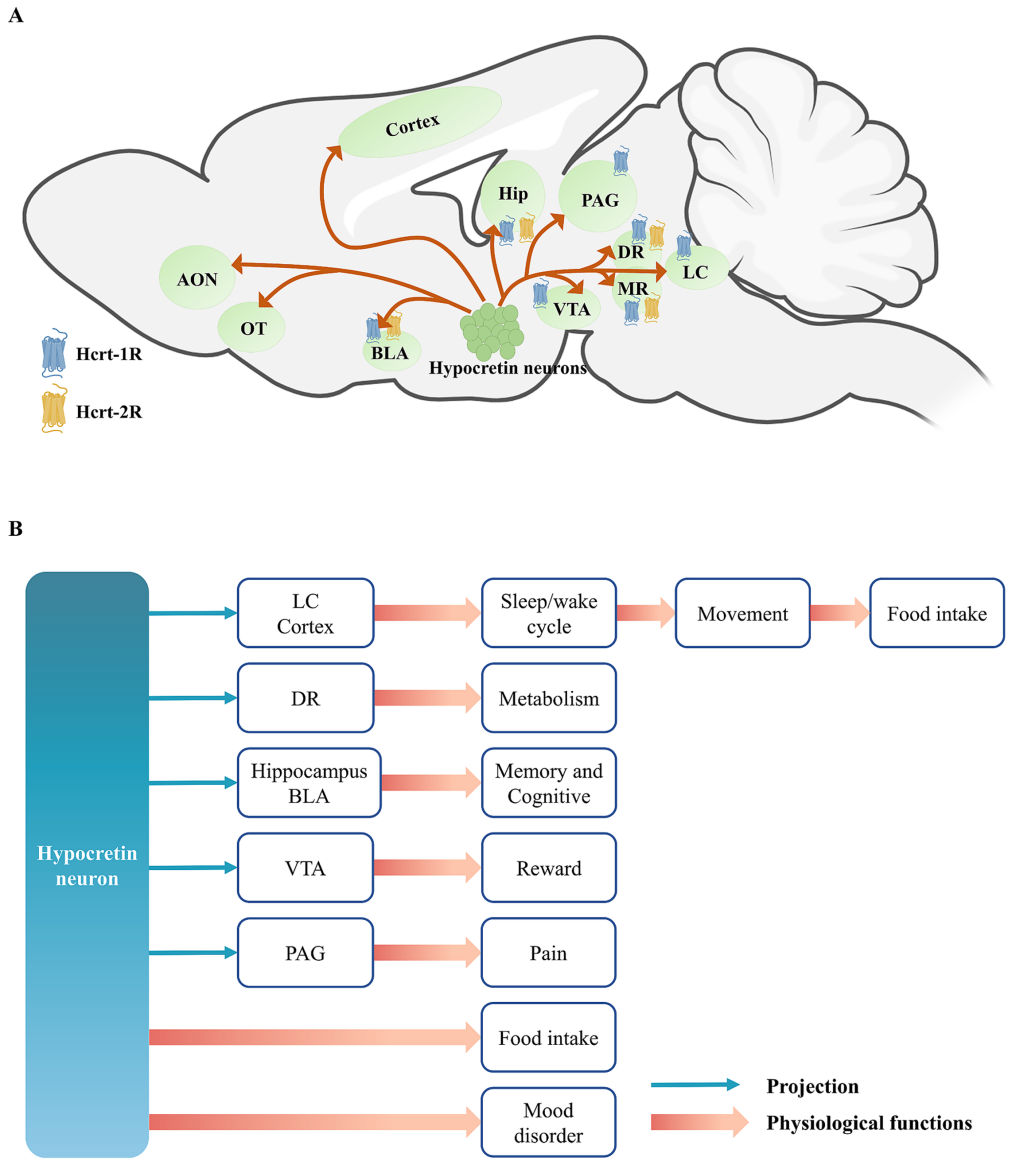
**(A)** The projections of hypocretin neurons and the distribution of hypocretin receptors. **(B)** The projections of hypocretin neurons and it’s regulated physiological functions. Hypocretin neurons exhibit extensive projections throughout the brain and participate in a variety of physiological functions, including the maintenance of the wakefulness-sleep system, food intake and energy metabolism, and emotional regulation, through two pathways: directly modulating the activity of downstream neurons or indirectly via the action of hypocretin peptides on their receptors. Among these, the regulation of wakefulness by hypocretin neurons can enhance the locomotor activity of animals, thereby driving them to forage for food, indirectly leading to food intake. AON, anterior olfactory nucleus; OT, olfactory tubercle; BLA, basolateral amygdala; Hip, hippocampus; PAG, periaqueductal gray; VTA, ventral tegmental area; DR, dorsal raphe; MR, medial raphe; LC, locus coeruleus.

Understanding the projection patterns of hypocretin neurons and the distribution of their receptors is essential to clarifying the physiological functions mediated by this system. In the following sections, we review these projections across major brain regions—the cortex, subcortex, and brainstem—and outline the functions supported by each pathway (Figure 1).

Hypocretin neurons project to multiple regions of the cerebral cortex, extending anteriorly to the anterior olfactory nucleus (AON) and the olfactory tubercle (OT) (Ahasan et al., 2024), and modulate odor-guided attractive and aversive behaviors by acting on the Hcrt-1R located in the OT. It also projects to the prefrontal cortex (PFC), motor cortex, and sensory cortex (Zolnik et al., 2020). These projection pattern regulates animal emotion and arousal states (Dong and Feng, 2018; Liu et al., 2024; Messore et al., 2025). It also enhances alertness and attention in animals, and enables to rapidly modulate cortical activity states in response to environmental stimuli, thereby supporting complex cognitive and behavioral responses (Karimi et al., 2019). Karimi et al. (2019) and Ahasan et al. (2024) primarily investigated the role of HCRT-1R in the prefrontal cortex (PFC) and orbitofrontal cortex (OFC) in decision-making. However, recent evidence indicates that HCRT-2R is the predominant receptor subtype in the cortex (Mir et al., 2024). Additionally, Liu et al. (2024) reported that blocking either HCRT-1R or HCRT-2R in the medial prefrontal cortex (mPFC) alleviates anhedonia induced by unpredictable chronic mild stress. Collectively, these findings suggest that hypocretin signaling in the cortex is both functionally significant and mechanistically complex, warranting further investigation.

Hypocretin neurons project to several subcortical nuclei, with their projections to the hippocampus primarily targeting the CA1 region and the dentate gyrus (DG), and those to the amygdala predominantly innervating the basolateral amygdala (BLA) (Barbosa et al., 2023). These projections are capable of modulating emotion and memory formation, particularly emotion-related memory, such as fear learning and memory, and social stress learning, by activating both Hcrt-1R and Hcrt-2R (Yaeger et al., 2022a; Yaeger et al., 2022b; Chen et al., 2024). This suggests that the hypocretin system may modulate emotion by influencing the formation or consolidation phase of memory. Furthermore, hypocretin neurons also project to the ventral tegmental area (VTA) of the midbrain, modulating the activity of dopaminergic neurons and thereby playing a significant role in reward and addictive behaviors (Harris et al., 2005; Kalló et al., 2022; Zamanirad et al., 2023; Mohammadkhani et al., 2024).

In the brainstem, the serotonergic neurons of the dorsal raphe (DR) express both Hcrt-1R and Hcrt-2R, and are involved in regulating systemic glucose homeostasis (Xiao et al., 2021), maintaining normal sleep architecture (Seifinejad et al., 2020), and influencing cataplexy (Hasegawa et al., 2017). PAG in the midbrain is a key component of the classical descending pain inhibitory system. Hypocretin can also act on the PAG to modulate pain-related behaviors. Specifically, hypocretin exerts analgesic effects, and activation of either Hcrt-1R or Hcrt-2R can produce analgesia (Azhdari Zarmehri et al., 2011; Lee et al., 2016).

LC is the brain region that receives the densest projections from hypocretin neurons (Jiao et al., 2025). Hypocretin can enhance the firing frequency of noradrenergic neurons in the LC by acting on Hcrt-1R, thereby promoting wakefulness (Mieda et al., 2013). Moreover, the projections of hypocretin in the LC are also involved in modulating conditioned fear learning, further indicating that hypocretin neurons are closely related to learning and memory processes (Soya et al., 2017).

## The role of hypocretin in sleep and arousal system

Earlier studies from the late 20th century, it had identified that hypocretin neurons in the lateral hypothalamus play a crucial role in maintaining wakefulness in humans, rodents, and canines. Mutations in hypocretin neurons or their receptors can lead to symptoms akin to narcolepsy, such as cataplexy and sleep fragmentation. Conversely, the administration of exogenous hypocretin has been shown to reverse these symptoms (Peyron et al., 1998; Chemelli et al., 1999; Lin et al., 1999; Hara et al., 2001; Mochizuki et al., 2004; Branch et al., 2016; Ishikawa et al., 2025).

Narcolepsy is a chronic neurological disorder marked by uncontrollable excessive daytime sleepiness (EDS) and abnormal regulation of rapid eye movement (REM) sleep. In both humans and rodents, the normal sleep–wake cycle comprises three primary states: wakefulness, non-rapid eye movement (NREM) sleep, and REM sleep. Under typical physiological conditions, sleep architecture follows a sequential pattern—transitioning from wakefulness to NREM sleep and then to REM sleep. This cyclical progression is essential for maintaining sleep consolidation and regulating arousal state (Ohayon et al., 2004; Siegel, 2009). However, this orderly progression is disrupted in individuals with narcolepsy, particularly those with type 1 narcolepsy (NT1). These patients often bypass NREM sleep and enter REM sleep directly upon falling asleep—a phenomenon known as sleep-onset REM episodes (SOREM), which is frequently accompanied by cataplexy (Mayà et al., 2023; Feng et al., 2024). Cataplexy is a pathognomonic feature characterized by sudden loss of muscle tone triggered by emotional stimuli. These clinical observations have promoted extensive investigation into the neurobiological basis of NT1. Subsequent research has revealed that type 1 narcolepsy (NT1) is primarily caused by the selective loss of hypocretin-producing neurons in the lateral hypothalamus. Supporting this, postmortem analyses have demonstrated approximately 90% reduction in these neurons. Furthermore, cerebrospinal fluid (CSF) levels of hypocretin-1 (≤110 pg./mL) are typically undetectable in NT1 patients, providing a reliable biomarker for diagnosis (Scammell, 2015; Mahoney et al., 2019).

Since its discovery, the role of hypocretin in sleep–wake regulation has been extensively investigated. Early research quantified hypocretin neuron activity across sleep stages using Fos expression (Estabrooke et al., 2001), while more recent techniques, such as *in vivo* calcium imaging, have provided detailed insights into their functional dynamics (Ito et al., 2023). These studies consistently show that hypocretin neurons are most active during wakefulness, display reduced activity during REM sleep, and fire intermittently during NREM sleep (Lee et al., 2005; Mileykovskiy et al., 2005). Loss or inhibition of hypocretin neurons results in reduced wakefulness and abnormal REM sleep architecture, which align closely with the clinical features observed in NT1 patients (Chemelli et al., 1999). Further investigations have revealed that LC, which receives the most densest projections from hypocretin projections, plays a key role in mediating these effects. Hypocretin directly increases the firing rate of noradrenergic (NE) neurons within the LC (Hagan et al., 1999; Carter et al., 2013). And inhibition of the LC abolishes hypocretin-induced arousal, confirming its critical role as a downstream effector in sleep–wake transitions (Bourgin et al., 2000; Carter et al., 2012; Carter et al., 2013). Other components of the ascending reticular activating system—including the tuberomammillary nucleus (TMN), laterodorsal tegmental nucleus (LDT), and pedunculopontine tegmental nucleus (PPT), also express hypocretin receptors to varying degrees, collectively contributing to the regulation of transitions between wakefulness, NREM, and REM sleep (Shouse and Siegel, 1992; Marcus et al., 2001; Xi et al., 2001; Mieda et al., 2011).

In addition to their excitatory effects, hypocretin neurons can also promote wakefulness through a feedforward inhibitory mechanism: they activate GABAergic interneurons within the ventrolateral preoptic nucleus (VLPO), which in turn inhibit sleep-promoting VLPO neurons (De Luca et al., 2022). In contrast, a distinct subpopulation of hypocretin neurons that project to the sublaterodorsal nucleus (SLD) displays REM sleep-specific activity. Optogenetic stimulation of these SLD-projecting neurons suppresses muscle tone to maintain REM sleep, whereas their inhibition shortens REM duration (Feng et al., 2020). Further evidence indicates that the activity of hypocretin neurons targeting the SLD is positively correlated with the post-inter-REM interval, suggesting that this pathway relieves REM sleep pressure and helps prevent sleep-onset REM (SOREM) episodes (Feng et al., 2024). These findings highlight a functional dichotomy within the hypocretin system: while overall hypocretin neuron activity appears relatively consistent across sleep stages, distinct subpopulations exert opposing effects—some facilitating wakefulness via VLPO inhibition, others stabilizing REM sleep through SLD engagement. This specialization enables the dynamic regulation of sleep–wake transitions in response to physiological demands.

In parallel to sleep regulation, hypocretin signaling has been increasingly implicated in metabolic processes as well. Although weight gain is not a diagnostic criterion for NT1, many patients typically experience weight gain. This occurs despite a lower basal metabolic rate and reduced caloric intake compared to healthy individuals (Lammers et al., 1996; Schuld et al., 2000; Hara et al., 2001; Poli et al., 2009). These observations suggest that the hypocretin system integrates arousal states with whole-body energy homeostasis, thereby regulating internal physiological balance (Chabas et al., 2007).

## The role of hypocretin in food regulation and energy metabolism

Hypocretin (also known as orexin, from the Greek “orexis” meaning appetite) plays a crucial role in feeding behavior regulation, underscoring the dual nomenclature of this neuropeptide system. As early as 1998, studies demonstrated that intracerebroventricular injection of either Hcrt1 or Hcrt2 significantly increased food intake in rats. Moreover, the mRNA levels of hypocretin were significantly elevated in the fasted state (Sakurai et al., 1998). Complementary findings showed that administration of hypocretin receptor antagonists into the cerebral ventricles of fasted rats led to a reduction in food consumption, further supporting its orexigenic role (Haynes et al., 2000; Yamada et al., 2000; Ishii et al., 2005). Further evidence indicates that hypocretin neurons are closely associated with anticipatory feeding behavior. In rats conditioned to a regular feeding schedule, hypocretin neurons were significantly activated in response to either the expectation of food or the visual presentation of food alone (Johnstone et al., 2006). In mice, fiber photometry studies revealed that hypocretin neuron activity rapidly declined upon initiation of feeding and remained suppressed during the feeding process, returning to an active state after feeding ceased (González et al., 2016). This suggests that food-seeking (foraging) and consummatory behaviors are mediated by distinct neural mechanisms, with hypocretin neurons being essential for the appetitive phase and reactivating whenever nutritional demand recurs.

More evidence supports that hypocretin regulates feeding behavior with more intricate mechanisms. Activation of hypocretin neurons has been shown to increase feeding while simultaneously increasing locomotion and exploratory actions such as running and climbing (Karnani et al., 2020). Notably, these behaviors occur independently of the vagal afferent pathway and are driven by direct neural mechanisms involving hypocretin signaling (Viskaitis et al., 2022). This adaptive behavior may represent an evolutionary mechanism, encouraging foraging when food is scarce, thereby enhancing survival by ensuring adequate energy acquisition.

Energy homeostasis depends on the precise balance between caloric intake and energy expenditure. As early as 1998, Lubkin and Stricker–Krongrad first demonstrated that intracerebroventricular administration of hypocretin-1 (hcrt1), but not hcrt2, increased oxygen consumption during the active phase in mice, suggesting hcrt1-specific modulation of basal metabolic rate (BMR) (Lubkin and Stricker-Krongrad, 1998). A major component of energy expenditure is thermogenesis mediated by brown adipose tissue (BAT), which produces heat via non-shivering thermogenesis driven by mitochondrial uncoupling protein 1 (UCP1) (Nowack et al., 2017; Yau and Yen, 2020). In 2011, Tupone et al. showed that microinjection of HCRT-1 into the rostral raphe pallidus (rRPa)—a key site for sympathetic premotor control of BAT—significantly enhanced BAT sympathetic outflow and thermogenic activity (Tupone et al., 2011). In contrast, earlier findings by Yoshimichi et al. (2001) reported that although third-ventricular administration of HCRT-1 elevated core body temperature in rats, it did not increase *Ucp1* mRNA expression, a molecular marker of BAT thermogenesis (Yoshimichi et al., 2001). Further insights from Sellayah and colleagues revealed that genetic ablation of hypocretin neurons in mice fed a high-fat diet led to a threefold increase in weight gain compared to controls and Hypocretin deficiency was also associated with reduced lean-mass energy expenditure and physical activity in both chow-fed and high-fat-fed mice, though reduced oxygen consumption was observed only in the latter (Sellayah et al., 2011; Sellayah and Sikder, 2012). Moreover, hypocretin-deficient mice exhibited exaggerated drops in core temperature during cold exposure and impaired BAT development, characterized by reduced *Ucp1* expression and compromised thermogenic capacity (Sellayah and Sikder, 2012; Sellayah et al., 2011). However, the absence of *Ucp1* upregulation following third-ventricular HCRT-1 injection suggests that the thermogenic effects of hypocretin may be mediated by co-released neurotransmitters such as glutamate or dynorphins (e.g., glutamate, dynorphins) (Yoshimichi et al., 2001; Sellayah et al., 2011). Collectively, these findings imply that hypocretin plays a more prominent role in promoting energy expenditure than in stimulating appetite, underscoring its critical function in metabolic regulation.

**Figure 2:**
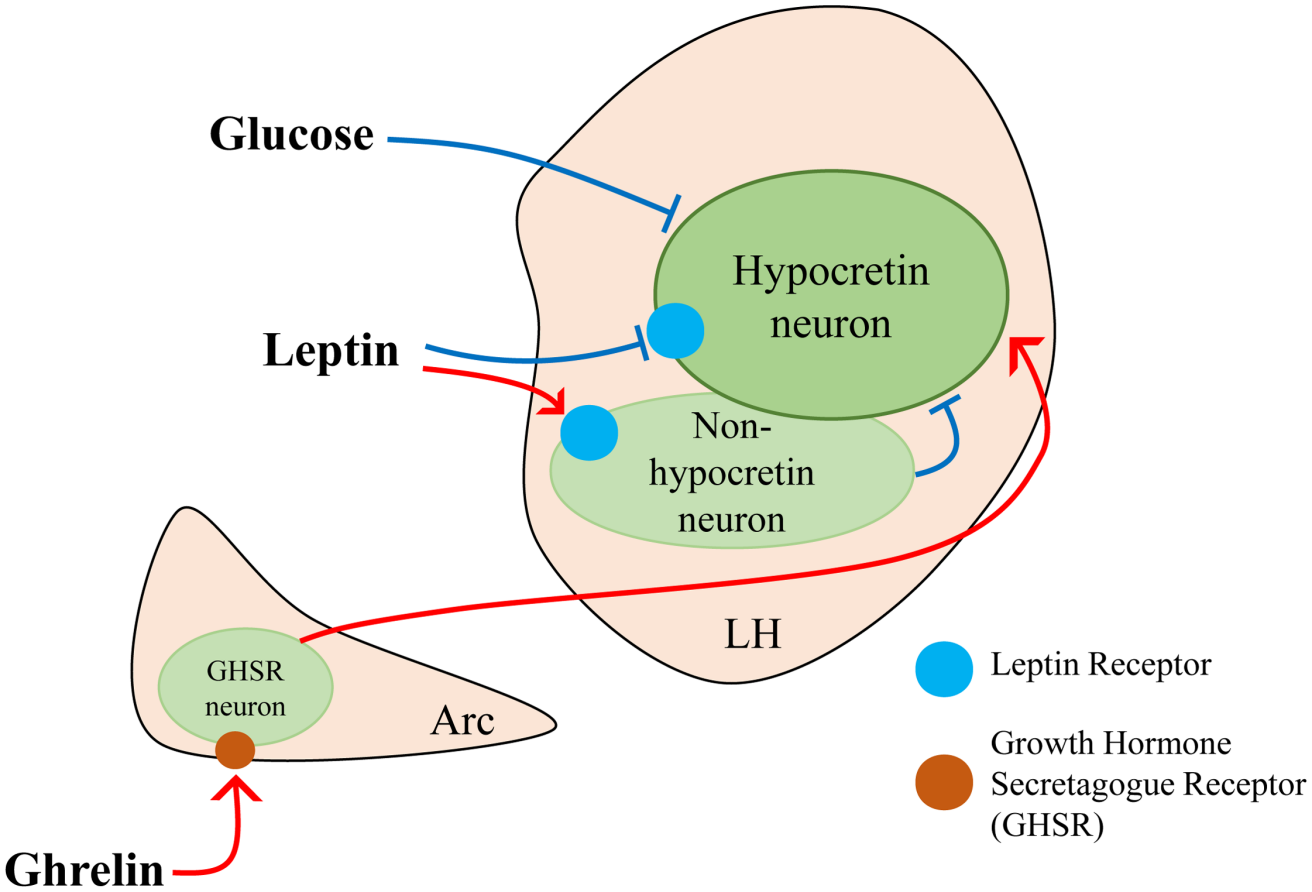
Glucose, leptin, and ghrelin jointly participate in the regulation of hypocretin neuron activity.

Beyond regulating food intake and energy expenditure, hypocretin neurons also play a critical role in nutrient sensing by rapidly detecting peripheral metabolic signals and adjusting behavioral responses accordingly. For instance, they are highly sensitive to fluctuations in blood glucose levels, exhibiting increased activity during hypoglycemia to stimulate appetite and restore energy balance (Yamanaka et al., 2003; Burdakov et al., 2005; Sheng et al., 2014; Viskaitis et al., 2024). Apart from glucose, ghrelin secreted by the stomach and leptin secreted by adipose tissue, bridge peripheral energy status with central nervous system regulation. Although hypocretin neurons lack ghrelin receptors (GHSRs), ghrelin indirectly influences them by activating NPY-expressing arcuate neurons and GHSR-positive, hypocretin-negative neurons in the lateral hypothalamus, forming local circuits that promote feeding (Howard et al., 1996; Hewson and Dickson, 2000; Nakazato et al., 2001; Nakazato et al., 2001; Burdakov and González, 2009; Romero-Picó et al., 2013; Medrano et al., 2018; Barrile et al., 2023; Teegala et al., 2023). During energy deficit, these two complementary pathways enable hypocretin neurons to register a peripheral decline in ghrelin and, consequently, to drive feeding behavior. Conversely, leptin—secreted by adipose tissue—acts through leptin receptors (LepRs), which are expressed in both hypocretin and non-hypocretin neurons (Friedman and Halaas, 1998). Moreover, non-hypocretin LH^LepR^ neurons, such as LH^LepRbNts^ neurons, can form synaptic connections with hypocretin neurons, with both populations contributing to the regulation of feeding behavior and energy homeostasis (Figure 2) (Håkansson et al., 1999; Louis et al., 2010; Leinninger et al., 2011).

These findings suggest that the role of the hypocretin system in energy metabolism extends beyond simply promoting appetite. Rather than acting in a unidirectional manner, hypocretin dynamically responds to physiological states, balancing energy intake and expenditure to maintain metabolic homeostasis. Understanding its function, therefore, requires consideration of the broader physiological context rather than reductionist interpretations. Importantly, the hypocretin system sits at the intersection of sleep regulation and metabolic control. Given the well-established link between sleep disruption and metabolic dysfunction, a key question arises: how does hypocretin coordinate these two homeostatic domains? Sleep restriction or deprivation has been shown to promote hyperphagia, particularly increasing the intake of high-fat, calorie-dense foods (Tajiri et al., 2023). This is partly driven by hormonal changes, including decreased leptin and increased ghrelin levels, which enhance hunger (Van Cauter et al., 2008; Gomes et al., 2023). Notably, during extended wakefulness, hypocretin neuron activity remains elevated to maintain arousal, a state that also facilitates increased, and often hedonic, food consumption (Adamantidis and de Lecea, 2008; Almeida Rojo et al., 2025).

This phenomenon is well-documented in human studies. For example, shift work and nighttime light exposure disrupt circadian rhythms and are associated with increased obesity risk. Prolonged disturbances in sleep architecture can promote unhealthy eating patterns, thereby increasing the likelihood of chronic conditions such as obesity and diabetes (Stenvers et al., 2019). Notably, many patients with NT1 exhibit obesity despite reduced caloric intake, suggesting that hypocretin neurons integrate arousal–sleep regulation with energy homeostasis. However, the direct role of the hypocretin system in linking sleep–wake states to metabolic control remains insufficiently explored and warrants well-designed investigation.

## The role of hypocretin in mood disorders

In daily life, emotional states often influence eating behavior—for example, one might say, “Let us go have a big meal” to celebrate a joyful occasion, or “eat some sweets to cheer up” when feeling upset. These expressions reflect hedonic feeding, a form of food intake driven not by metabolic need but by the pursuit of pleasure. Unlike homeostatic feeding, hedonic feeding is regulated by reward-related neural circuits, particularly those involving dopaminergic signaling, and is closely associated with emotional regulation.

In addition to their projections to the nucleus accumbens (NAc) and ventral tegmental area (VTA), which facilitate reward-related behaviors, hypocretin neurons also innervate the amygdala—thereby contributing to the regulation of emotional responses. This anatomical connectivity underscores the role of the hypocretin system in integrating reward processing with affective modulation. In mice, hypocretin neuron activity has been shown to correlate with stress resilience, and activation of these neurons enhances coping behaviors under stress (Kim et al., 2023). Similarly, targeted activation of lateral hypothalamus hypocretin neurons alleviated anxiety-like behaviors induced by social defeat stress (Wang et al., 2021). These findings underscore the relevance of hypocretin signaling in modulating stress, anxiety-related behaviors in animal models.

Major depressive disorder (MDD) is the most common type of mood disorder. Some theories suggest that MDD represents a maladaptive response to chronic stress and anxiety (Buntrock et al., 2024; Cui et al., 2024; Yang et al., 2025). Although only a few animal studies have investigated the relationship between the hypocretin system and MDD, there have some clinical investigations have demonstrated elevated levels of plasma hypocretin-1 in MDD patients, whereas others have found no significant association between plasma hypocretin-1 levels and the presence of depression (Tsuchimine et al., 2019; Akça et al., 2021; Li et al., 2021; Chen et al., 2024). These inconsistencies may stem from methodological factors variability in sampling times, limited sample sizes, and inter-individual physiological differences. Importantly, plasma hypocretin concentrations may not reliably reflect central hypocretin neurons’ functionality. In contrast, cerebrospinal fluid (CSF) offers a more direct assessment of central hypocretin activity. However, the invasive nature of lumbar puncture has limited the scope and scale of CSF-based research.

Notably, studies have indicated that in patients with MDD, those exhibiting suicidal behavior display significantly reduced levels of hypocretin-1 in the CSF (Brundin et al., 2007). Although these findings lack a healthy control group for comparison, they nonetheless suggest a possible link between hypocretin deficiency and suicidality. It is noteworthy that a study published in 2011 reported no significant difference in CSF hypocretin-1 levels between 17 patients with MDD and 10 healthy controls. However, given the substantial age difference between the groups (patients: 51.3 ± 16.2 years; healthy controls: 36.4 ± 11.8 years) and the fact that all MDD patients had a history of medication use, the question of whether there is a difference in CSF hypocretin-1 levels between MDD patients and healthy individuals remains to be further investigated (Schmidt et al., 2011).

Although the precise mechanisms underlying the role of the hypocretin system in MDD remain unclear, emerging pharmacological evidence points to the potential utility of hypocretin receptor antagonists in alleviating depressive symptoms. A recent meta-analysis of five randomized clinical trials involving 498 participants assessed the efficacy of various HCRT-2R antagonists, Seltorexant, one used the HCRT-1/2R antagonist, Filorexant, and another employed the HCRT-1/2R antagonist Suvorexant. The results indicated that Seltorexant holds the most promise for therapeutic efficacy in treating MDD, Suvorexant showed a trend toward improvement, while Filorexant yielded less favorable outcomes (Meshkat et al., 2025). These findings highlight the potential of targeting the hypocretin system for MDD treatment. Although larger, standardized trials are required to confirm these effects and benchmark them against conventional antidepressants and novel therapeutic options.

Clinical studies have shown that sleep disturbances can lead to mood disorders such as anxiety or depression (Dinges et al., 1997; Tomaso et al., 2021). The underlying neural mechanisms may involve heightened amygdala reactivity and reduced regulatory capacity of the prefrontal cortex due to sleep loss (Ben Simon et al., 2020), making individuals more susceptible to negative emotions. Additionally, sleep deprivation can lead to elevated cortisol levels, keeping the body in a prolonged “fight or flight” stress state (Nollet et al., 2020).

To date, limited research has explored the role of the hypocretin system in mood disorders associated with sleep deprivation. Notably, a study by Allard et al. (2007) investigated this relationship using the Wistar-Kyoto (WKY) rat model of depression, which naturally exhibits elevated REM sleep. Employing the small-platform-over-water method to induce REM sleep deprivation, the authors observed a 20% increase in the number of hypocretin neurons in WKY rats. Interestingly, the large-platform control group showed an even greater increase of 31% (Allard et al., 2007). We speculate that this may help explain why related studies are scarce. In animal models, sleep deprivation primarily induces stress responses and metabolic disturbances (e.g., activation of the HPA axis), and its effects on hypocretin neurons are far more pronounced than its impact on mood disorders.

Patients with narcolepsy often experience certain psychiatric symptoms, such as anxiety, depression, and hallucinations (Billiard et al., 2006; Scammell, 2015). This may be partly due to the loss of hypocretin neurons, and partly due to mood disorders resulting from long-term cataplexy and disrupted sleep patterns.

## Hypocretin as pharmacological target in the treatment of narcolepsy, obesity, and mood disorders

### Hypocretin as pharmacological target in the treatment of narcolepsy

Narcolepsy is directly associated with a severe deficiency in the hypocretin system. Patients with NT1 exhibit significantly reduced levels of hypocretin-1 in cerebrospinal fluid (typically ≤110 pg./mL). The management of narcolepsy requires a combination of behavioral interventions and pharmacological treatments. In terms of behavioral approaches, regular napping and a consistent sleep pattern are beneficial in reducing EDS. Regarding pharmacological treatment, there is currently no definitive etiological therapy in clinical practice. The focus is primarily on symptomatic treatment. For instance, for the management of EDS, the first-line medication is pitolisant, a novel histamine H3 receptor antagonist/inverse agonist. Pitolisant has also been shown to significantly improve cataplexy, a condition characterized by sudden muscle weakness triggered by strong emotions. Other medications, such as modafinil, which can enhance hypocretin-dependent histaminergic neurotransmission in the tuberomammillary nucleus of the thalamus, are also used. The clinically recommended anti-cataplectic medications mainly include pitolisant, sodium oxybate, and venlafaxine, among others (Billiard et al., 2006; Dauvilliers et al., 2013; Szakacs et al., 2017; Abad, 2019).

The aforementioned treatments primarily alleviate symptoms by modulating neurotransmitter systems, yet they fail to address the underlying cause: dysfunction of the hypocretin system. At this stage, narcolepsy medications targeting the hypocretin system are still confined to animal and clinical research stages. Peripheral administration of hypocretin is limited by its poor ability to cross the blood–brain barrier. However, studies have shown that intranasal drug delivery can bypass the blood–brain barrier and directly act on the central nervous system (Dhuria et al., 2009; Calva and Fadel, 2020). Animal research has demonstrated that intranasal administration can reverse cognitive deficits induced by sleep deprivation in non-human primates, with effects superior to those of intravenous administration (Deadwyler et al., 2007). Nevertheless, research on intranasal drug delivery for the treatment of narcolepsy is virtually non-existent at present. On the other hand, peptide-based drugs cannot be taken orally and are easily degraded (Muttenthaler et al., 2021). Similarly, small-molecule non-peptide hypocretin receptor agonists are under development and have entered early-stage clinical trials (Fujimoto et al., 2022; Ishikawa et al., 2023). However, large-scale, long-term clinical trials in humans are still lacking, and further research is required to comprehensively evaluate their efficacy and safety. Although the drugs currently used clinically to treat narcolepsy have limitations in terms of side effects and therapeutic efficacy, they have established a mature usage experience in clinical practice. Therefore, drugs targeting the hypocretin system need to demonstrate more significant advantages in order to replace the existing treatment paradigms.

To date, numerous selective and non-selective hypocretin receptor antagonists have been developed, such as suvorexant, Lemborexant, and daridorexant (Cox et al., 2010; Murphy et al., 2017; Dauvilliers et al., 2020), which are often used in the treatment of insomnia (Table 1). In contrast to the extensive development of antagonists, research on hypocretin receptor agonists for the treatment of narcolepsy is relatively limited. The most advanced in development is oveporexant (TAK-861), which is currently in Phase III clinical trials. The New England Journal of Medicine published its Phase II clinical trial study in March 2025. The results indicated that oveporexant, as a selective hypocretin-2 receptor (HCRT-2R) agonist, significantly improved wakefulness, EDS, and cataplexy symptoms in patients with NT1, without observed hepatotoxicity (Dauvilliers et al., 2025). In addition to oveporexant, other selective HCRT-2R agonists such as ALKS-2680, TAK-360, and ORX-750 are currently in Phase II or Phase III clinical trials (Table 1).

**Table 1:** Drugs targeting the hypocretin receptor.

Action	Target	Drug	Drug highest phase	Active indication	Clinical trials	References
Agonist	Hcrt-2R	Oveporexton (TAK-861)	Phase 3	Narcolepsy, cataplexy	NCT06505031 (Active); NCT06470828 (Active); NCT05816382 (Active)	Mitsukawa et al. (2024), Dauvilliers et al. (2025)
		ALKS-2680 (RDC-2641)	Phase 3	Narcolepsy, cataplexy	NCT06358950 (Active); NCT06555783 (Active); NCT06843590 (Active); NCT06767683 (Active)	Yee et al. (2023), Grunstein et al. (2025), Modi et al. (2025)
		TAK-360	Phase 2	Narcolepsy	NCT06812078 (Active); NCT06952699 (Active); NCT006812078 (Active)	
		ORX-750	Phase 2	Narcolepsy	NCT06752668 (Active)	Hartman et al. (2025)
		E-2086	Phase 1	Cataplexy	NCT06462404 (Completed); NCT06481488 (Completed)	
		DSP-0187 (JZP-441)	Phase 1	Narcolepsy	NCT06961266 (Active); NCT05720494 (Completed); NCT05651152 (Completed)	
	Hcrt-1R	INDV-2000 (C4X-3256)	Phase 2	Opioid abuse	NCT06384157 (Active); NCT04976855 (Completed); NCT05694533 (Completed); NCT04413552 (Completed)	
		AZD-4041	Phase 1	Opioid abuse	NCT05587998 (Completed); NCT05233085 (Completed); NCT04076540 (Completed)	
Antagonist	Hcrt-2R and Hcrt-1R	Daridorexant hydrochloride (ACT-541468, DORA, Quviviq, SIM-0808)	Approved	Sleep disorders, PTSD	NCT05948540 (Active)	Di Marco et al. (2023), Kunz et al. (2023), Nie and Blair (2023), Williams and Rodriguez-Cué (2023)
Antagonist	Hcrt-2R and Hcrt-1R	Lemborexant (Dayvigo, E-2006, LEM-10, LEM-5)	Approved	Sleep disorders, AD, OSAS		Beuckmann et al. (2017), Murphy et al. (2017), Kärppä et al. (2020), De Crescenzo et al. (2022), Kushida et al. (2025)
		Suvorexant (Belsomra, L-001958419, K-4305)	Approved	Sleep disorders, AD, OSAS		Cox et al. (2010), Herring et al. (2012), Sun et al. (2013), Kishi et al. (2024)
		Fazamorexant (YZJ-1139)	NDA/BLA	Sleep disorders, central nervous system disease	NCT06975514 (Active); NCT05525637 (Active); NCT06680505 (Completed)	
		Vornorexant (ORN-0829, TS-142)	Phase 3	Sleep disorders	NCT05819710 (Completed); NCT05453136 (Completed)	Futamura et al. (2020), Kambe et al. (2023)
	Hcrt-2R	Seltorexant (JNJ-42847922, JNJ-7922, JNJ-922, MIN-202)	Phase 3	MDD, sleep disorders, anxiety disorders, OSAS	NCT06559306 (Active); NCT05307692 (Completed); NCT05106153 (Completed)	Recourt et al. (2019), Savitz et al. (2021), Jha (2022), Mesens et al. (2025)
		HS-10506	Phase 2	Sleep disorders, depressive disorders	NCT05953506 (Active); NCT06279286 (Active)	
		BrP-01096	Phase 1	Sleep disorders	CTR20241617 (Active)	
	Hcrt-1R	CVN-766	Phase 1	Binge-eating disorder, obesity, schizophrenia	NCT05105243 (Completed)	Glen et al. (2024)

NDA, New Drug Application; BLA, Biologics License Application; PTSD, Post-Traumatic Stress Disorder; AD, Alzheimer’s Disease; OSAS, Obstructive Sleep Apnea Syndrome; MDD, Major Depressive Disorder.

### Hypocretin as pharmacological target in the treatment of obesity

Although narcolepsy frequently co-occurs with obesity (BMI ≥ 28), the mechanisms underlying this association remain poorly understood. At present, among the limited pharmacological agents targeting the hypocretin system for obesity treatment, only mazindol has received clinical approval. Mazindol is a multifunctional compound that inhibits the reuptake of norepinephrine, dopamine, and serotonin, thereby modulating mood, alleviating symptoms of depression and anxiety, and potentially promoting weight loss by influencing appetite and energy expenditure. Notably, mazindol also activates the HCRT-2 receptor, positioning it as a candidate therapeutic agent for both obesity and narcolepsy.

### Hypocretin as pharmacological target in the treatment of mood disorders

As previously mentioned, the mechanisms by which the hypocretin system influences mood disorders need to be further investigated. To date, only the HCRT-2R antagonist seltorexant, which is currently in Phase III clinical trials, has been demonstrated to be effective in the treatment of major depressive disorder and anxiety disorders (Meshkat et al., 2025).

### Hypocretin as a therapeutic target still face many challenges

Despite the critical role of the hypocretin system in numerous physiological processes and its prominence as a promising target for drug development, several significant challenges remain. First, as a neuropeptide, hypocretin is highly susceptible to enzymatic degradation when administered peripherally and exhibits poor permeability across the blood–brain barrier. Current efforts to deliver hypocretin intranasally are still in the experimental stage Second, the hypocretin receptors—HCRT-1R and HCRT-2R—are G-protein-coupled receptors with complex structures and signaling mechanisms. Each receptor subtype mediates distinct downstream pathways, and achieving receptor selectivity remains a major obstacle in therapeutic design (Zhang et al., 2024; Chaki, 2025). Third, because the hypocretin system is involved in diverse physiological functions, off-target effects are difficult to avoid. For example, in the treatment of alcohol addiction, hypocretin-targeting agents may inadvertently disrupt sleep regulation (Moorman, 2018). In summary, while the hypocretin system represents a compelling target for therapeutic intervention, its clinical application is still limited by pharmacological, structural, and safety-related challenges. Inconsistencies in clinical trial outcomes further highlight the need for continued research and innovation in this area.

## Discussion

### Future perspectives of hypocretin-related studies

The hypocretin (orexin) system has emerged as a central regulator of numerous physiological functions, including sleep–wake regulation, feeding behavior, energy metabolism, and emotion processing. While substantial progress has been made in elucidating its role-particularly in the pathogenesis of type 1 narcolepsy (NT1)-several critical knowledge gaps persist. Future research aimed at addressing these limitations hold promise for advancing both basic neuroscience and clinical therapeutics.

One unresolved area is the precise mechanisms underlying the co-occurrence of narcolepsy and obesity remain unclear. As number of studies have revealed, the hypocretin systems in the regulation of metabolism including promoting food seeking, elevating systemic glucose level, and the conditioned knockout of hypocretin receptors have showed improved glucose tolerance and body weight gain. Whereas, lacking of hypocretin neurons in NT1 model or patients induced certain amount of obesity phenotype. This inconsistency implies strong link between hypocretin system and other metabolism regulators which may be inhibited in the normal physiology status.

In parallel, regarding the role of hypocretin signaling in mood disorder, current studies have been limited to the hypocretin level in the disease model or patients, details mechanism explaining how the hypocretin system regulate mood behaviors by related neurocircuit or corresponding receptor system remain further explored. Additionally, standardizing methodologies including sample size, confounding factors (age, medication use and disease severity) in clinic is important for the consistent results. Nonetheless, evidence from both human and animal studies suggests the promise effect of hypocretin signaling to influence stress responses, anxiety-like behaviors and emotional resilience.

Pharmacological modulation of the hypocretin system remains an active area of investigation. While several dual hypocretin receptor antagonists (DORAs) have been approved for the treatment of insomnia, the development of hypocretin receptor agonists, potentially beneficial for conditions like NT1, is still in early stages. Major challenges include the difficulty of delivering peptide-based drugs across the blood–brain barrier and minimizing off-target effects. Alternative delivery strategies, such as intranasal administration or small-molecule agonists, may help overcome these obstacles. Moreover, long-term safety and efficacy data are needed to support the widespread clinical use of these agents.

To advance the field, future studies should prioritize the following: (1) elucidating the molecular and circuit-level mechanisms by which hypocretin neurons regulate metabolic and emotional homeostasis; (2) conducting large-scale, rigorously controlled clinical trials to clarify the role of hypocretin signaling in mood disorders; (3) developing and optimizing pharmacological agents that modulate the hypocretin system, including novel receptor agonists; and (4) leveraging interdisciplinary approaches—such as neuroimaging, optogenetics, and genetic models—to map the functional connectivity and plasticity of hypocretin circuits.

In conclusion, although much has been learned about the hypocretin system since its discovery, important questions remain unanswered. Addressing these challenges through innovative, integrative research will be essential for translating basic findings into effective treatments for a range of neurological, metabolic, and psychiatric disorders.
